# Isobavachalcone from *Angelica keiskei* Inhibits Adipogenesis and Prevents Lipid Accumulation

**DOI:** 10.3390/ijms19061693

**Published:** 2018-06-06

**Authors:** Hyejin Lee, Hua Li, Minson Kweon, Youngsook Choi, Min Jung Kim, Jae-Ha Ryu

**Affiliations:** 1 Research Center for Cell Fate Control and College of Pharmacy, Sookmyung Women’s University, 100 Chungparo 47-Gil, Yongsan-Gu, Seoul 04310, Korea; u9698115@naver.com (H.L.); cooldog227@hotmail.com (H.L.); minson-_-@nate.com (M.K.); 2 Research institute of women’s health, Sookmyung Women’s University, 100 Chungparo 47-Gil, Yongsan-Gu, Seoul 04310, Korea; k9701084@gmail.com; 3 Department of Biological Sciences, Sookmyung Women’s University, 100 Chungparo 47-Gil, Yongsan-Gu, Seoul 04310, Korea; minkim@sm.ac.kr

**Keywords:** *Angelica keiskei*, isobavachalcone, 3T3-L1 adipocyte, adipogenesis, clonal expansion, autophagy

## Abstract

We isolated isobavachalcone (IBC) from *Angelica keiskei* (AK) as an anti-obesity component. IBC dose-dependently inhibited 3T3-L1 adipocyte differentiation by down-regulating adipogenic factors. At the mitotic clonal expansion stage (MCE), IBC caused cell cycle arrest in G0/G1 with decreased expression of cell cycle-regulating proteins. IBC also inhibited autophagic flux by inducing intracellular accumulation of LC3B and SQSTM1/p62 proteins while decreasing expression levels of regulating factors for autophagy initiation. In parallel with the inhibition of adipocyte differentiation, IBC decreased intrahepatic fat deposits and rescued the liver steatosis in high fat cholesterol diet-fed zebrafish. In this study, we found that IBC isolated from AK suppresses mitotic clonal expansion and autophagy flux of adipocytes and also shows anti-obesity activity in a high cholesterol-diet zebrafish model by decreasing intrahepatic fat deposits. These results suggest that IBC could be a leading pharmacological compound for the development of anti-obesity drugs.

## 1. Introduction

Obesity is caused by excessive nutrition, a sedentary lifestyle, and genetic factors, resulting in energy imbalance. Obesity is associated with metabolic disorders such as diabetic mellitus, hypertension, cardiovascular disease, dyslipidemia, gallbladder disease, and cancer. According to an Organization for Economic Cooperation and Development (OECD) report in 2017, more than 50% of adults and 17% of children are obese or overweight in some countries, although global efforts have been made to prevent obesity [1].

Treatment for obesity consists of bariatric surgery and non-pharmacological treatment such as exercise, change in lifestyle, caloric intake limitation, and pharmacological treatment. When non-pharmacological treatment and bariatric surgery have unsatisfactory results, more promising and safe pharmacological treatments are needed to treat obesity. Although anti-obesity drugs including orlistat, lorcaserin, phentermine, and topiramate are currently available for weight loss, they might have side effects. Therefore, we have focused on discovering anti-obesity agents from natural products that can be less toxic and can be combined with conventional treatments [2].

The anti-obesity effect of reported natural products can be classified into three categories based on mechanism of action: (1) Reducing fat absorption; (2) regulating appetite; and (3) inhibiting white adipose tissue formation. Adipose tissue is composed of the connective tissue matrix, nerve tissue, and various cell types such as adipocytes, stromal vascular cells, endothelial precursor cells, immune cells, and mesenchymal stem cells. It plays an important role in energy homeostasis by regulating glucose and lipid metabolism [3]. Excessive size (hypertrophy) and number (hyperplasia) of adipocytes are concomitant with adipose tissue dysfunction, leading to obesity and obesity-associated diseases, including diabetes, hypertension, atherosclerosis, and cancer. Recent studies have demonstrated that several natural compounds can overcome obesity by regulating adipocyte formation and functions [2,4]. Berberine, resveratrol, curcumin, (−)-epigallocatechin gallate, and anthocyanins all exert anti-obesity activities by reducing adipocyte proliferation and differentiation in vitro and/or in vivo [2,5].

*Angelica keiskei* (AK) is known as ‘Sinsuncho’ in Korean, meaning the herb of eternal youth. It has been widely used to treat anemia, diabetes, hypertension, aging, and cancer in Asia. It contains therapeutic constituents such as coumarins, flavanones, and chalcones [6]. Several studies have reported the anti-osteoporotic, anti-diabetic, anti-melanogenic, anti-atherosclerotic, and anti-obesity potential of compounds that are also known as major ingredients of AK [7,8,9,10,11]. Among these compounds, chalcones (α,β-unsaturated ketones) have diverse derivatives as intermediates in the biosynthesis of bioactive flavonoids [2]. Although extensive studies have been conducted to discover the pharmacological potential of chalcones, molecular mechanisms of action and applicable in vivo data are still insufficient to explain the therapeutic potential of chalcones [12,13]. In this study, we isolated isobavachalcone (IBC) from AK, a chalcone constituent, and investigated its anti-obesity potential. IBC is known to possess anti-bacterial, anti-fungal, anti-cancer, anti-tubercular, and anti-oxidant activities in various research models [14]. Yang et al. reported that IBC has an inhibitory effect on adipogenesis [15]. However, its underlying mechanism remains unclear. Here, we report the effect of IBC on adipogenesis using 3T3-L1 preadipocytes and a high cholesterol-diet zebrafish model and the mechanisms of IBC action.

## 2. Results

### 2.1. Isobavachalcone (IBC) Inhibits Adipocyte Proliferation and Differentiation

**Figure 1:**
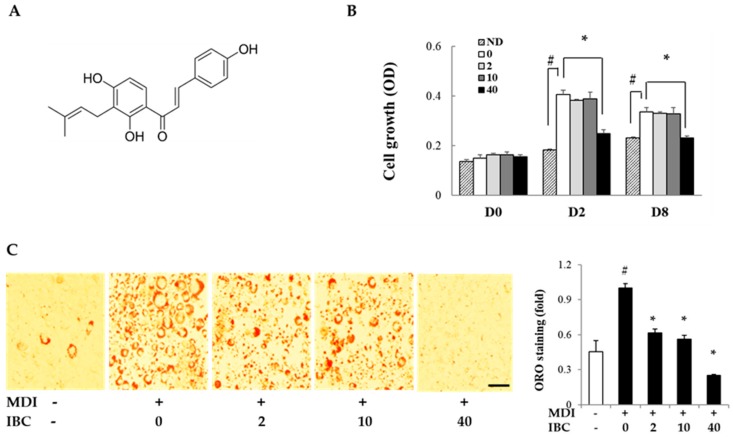
Structure of isobavachalcone (IBC) and effect of IBC on adipocyte growth and differentiation. (**A**) Structure of IBC isolated from *A. keiskei* (AK); (**B**) 3T3-L1 preadipocytes were differentiated with differentiation medium (MDI) in the presence or absence of IBC (0, 2, 10, or 40 μM). On differentiation day 0, 2, or 8 (D0, D2, or D8), cells were subjected to MTT assay; and (**C**) 3T3-L1 cells were differentiated with IBC at indicated concentration. Cells were stained with Oil Red O (ORO) on D8, and intracellular lipid contents were quantified as described in Materials and Methods. Adipocytes stained with ORO (magnification, 40×) were visualized by light microscopy. Scale bar = 100 μm. Data are means ± standard deviation (SD) of triplicate experiments. # *p* < 0.01 vs. control (no differentiation, ND); * *p* < 0.01 vs. MDI only.

IBC was isolated from the ethyl acetate soluble fraction of *A. keiskei* (AK) extracts and its structure was identified by spectroscopic analysis (Figure 1A) [6]. To determine the inhibitory effect of IBC on adipocyte proliferation, MTT assay was performed on different differentiation days: 0, 2, and 8. On D2 and D8, cell proliferation was increased by MDI treatment compared to the respective control treatment. IBC significantly inhibited cell proliferation by 38.6% (D2) and 31.0% (D8) compared to the differentiation medium (MDI) treatment (Figure 1B). In our preliminary experiments, we found that 48 h treatment of IBC showed no effect on cell viability of 3T3-L1 preadipocytes.

The anti-adipogenic effect of IBC was also determined in vitro by Oil Red-O (ORO) staining at D8 (Figure 1C). MDI treatment resulted in a large amount of intracellular lipid contents. However, the IBC dose dependently inhibited lipid accumulation. IBC at 40 μM resulted in the maximal inhibitory effect (75.0%) compared to MDI treatment.

Taken together, these results suggest that IBC can significantly inhibit cell proliferation during the mitotic clonal expansion (MCE) period of D0–D2 and adipocyte differentiation, implying that IBC can down-regulate the early phase of adipogenesis.

### 2.2. IBC Inhibits Mitotic Clonal Expansion via Cell Cycle Arrest

**Figure 2:**
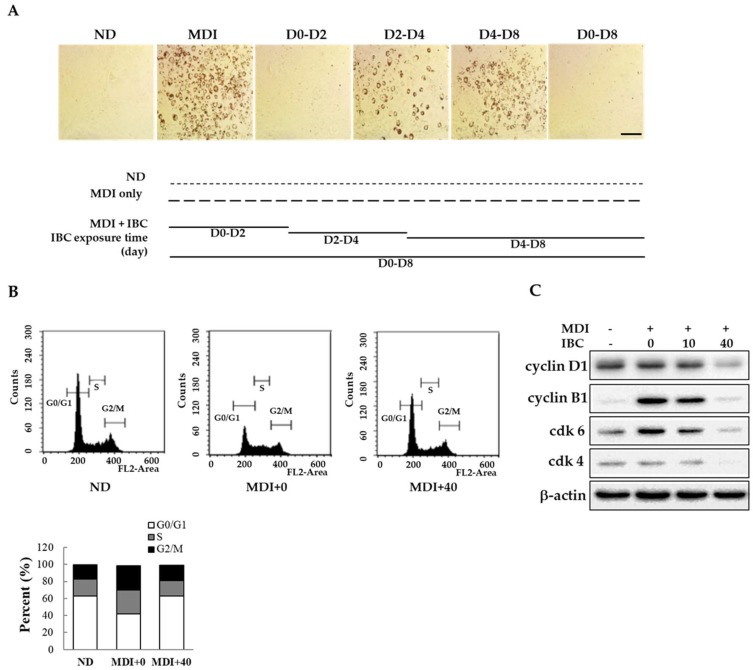
Inhibitory effect of IBC on mitotic clonal expansion. (**A**) Confluent 3T3-L1 preadipocytes were differentiated with MDI to induce differentiation in the presence or absence of IBC (40 μM). Cells were exposed to IBC during indicated differentiation periods. On D8, all groups were simultaneously subjected to ORO staining. ND, not-differentiated; MDI, differentiation medium. Scale bar = 100 μm; (**B**) Differentiation initiated adipocytes (D0) were treated with IBC for 48 h followed by flow cytometry; and (**C**) Cell lysates were prepared for Western blotting using indicated antibodies as described in Materials and Methods.

Adipogenesis has three distinct stages of differentiation: Early, intermediate, and late stages [16]. To determine the specific time point at which IBC acted on differentiation, 3T3-L1 preadipocytes were treated with 40 μM IBC at D0–D2, D2–D4, D4–D8, and D0–D8 periods in the presence of MDI. Intracellular lipid contents were then determined on D2, D4, or D8 of differentiation using ORO staining. As shown in Figure 2A, exposure to IBC during D0–D2 completely attenuated adipocyte differentiation based on decreased ORO staining. However, cells treated with IBC during the D2–D4 or D4–D8 period displayed a weak reduction in lipid contents. Thus, IBC-mediated suppression of adipogenesis occurred during the early stage of adipocyte differentiation.

Mitotic clonal expansion (MCE) is a mandatory process to enter the differentiation step. It occurs at the early stage of adipocyte differentiation. Confluent preadipocytes undergo about two rounds of cell cycle during the MCE stage. To determine whether MCE might be affected by IBC, confluent adipocytes were treated with MDI or a combination of MDI and IBC for 48 h and then subjected to flow cytometry analysis (Figure 2B). The percentage of cells in G0/G1 phase in the MDI treated group was 41.9% compared to 62.6% in untreated cells (not-differentiated, ND). On the contrary, IBC showed the percentage of cells in the G0/G1 phase (62.9%). In proliferating cells, certain cyclins and cyclin-dependent kinase (cdk) at specific times are essential for cell cycle progression. It is known that the cyclin D1-cdk4/6 complex plays a role in G0/G1-to-S phase transition [17]. Thus, we assessed protein levels of cyclin D1, cdk-4, and cdk-6 in IBC-treated adipocytes during the MCE stage. As shown in Figure 2C, the IBC dose dependently inhibited the protein expression levels of cyclin D1, cdk-4, and cdk-6 within 48 h after MDI stimulation. IBC accumulated cell numbers in the G0/G1 phase, resulting in decreased expression of cyclin B1 known to be involved in cell cycle progression. These data demonstrate that IBC can inhibit adipocyte differentiation by inducing G0/G1 phase arrest at the MCE stage.

### 2.3. IBC Reduces Gene Expression of Transcription Factors during Adipocyte Differentiation

**Figure 3:**
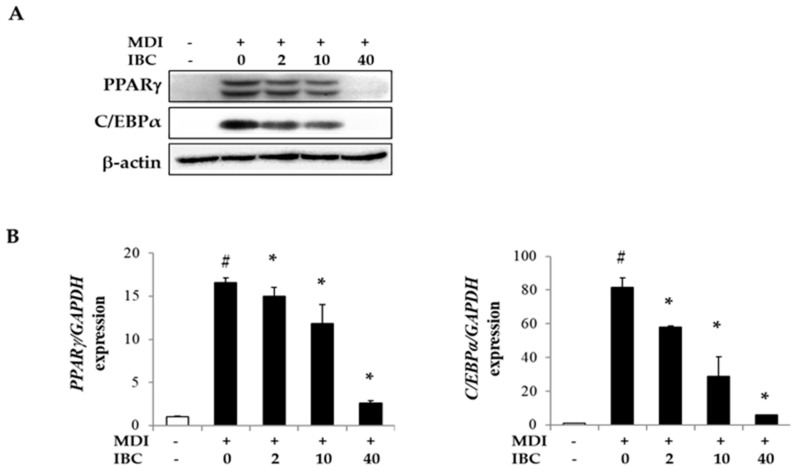
Inhibitory effect of IBC on expression of adipogenic transcription factors. 3T3-L1 adipocytes were differentiated with IBC at indicated concentrations. On D5, cells were harvested and lysed to perform Western blotting analysis (**A**) and quantitative real time PCR (qPCR) (**B**). Data are presented as means ± SD of triplicate experiments. # *p* < 0.01 vs. control; * *p* < 0.01 vs. MDI only.

Peroxisome proliferator activated receptor subtype γ (*PPARγ*) and CCAAT/enhancer binding protein-α (*C/EBPα*) are important transcription factors for adipogenesis. To understand the underlying mechanism of adipogenesis suppression by IBC, we measured mRNA and protein levels of adipogenic transcription factors by quantitative real time PCR (qPCR) and Western blotting analysis (Figure 3). Protein levels of PPARγ and C/EBPα were found to be dramatically decreased by 85.5% and 97.3%, respectively, in IBC (40 μM) treated cells. Levels of *PPARγ* and *C/EBPα* mRNA were also decreased by IBC compared to those in MDI only treated cells. These results indicate that IBC can modulate adipocyte differentiation by suppressing the expression of adipogenic transcription factors.

### 2.4. IBC Decreases Gene Expression of Lipid Metabolism-Regulating Factors during Adipocyte Differentiation

**Figure 4:**
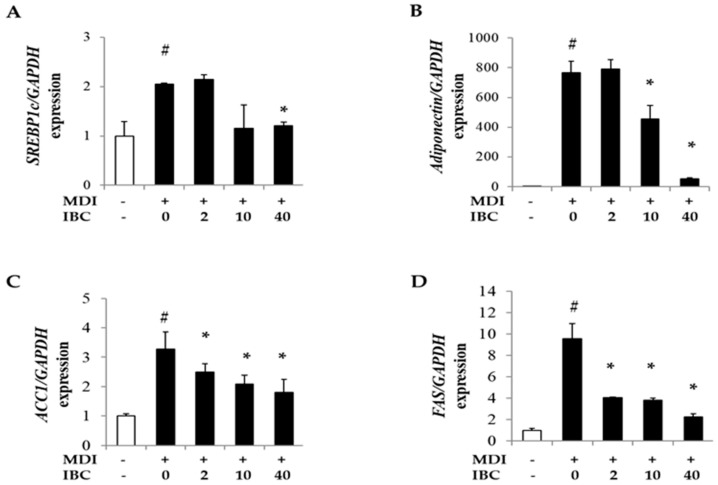
Effect of IBC on levels of lipid metabolism related genes. 3T3-L1 adipocytes were differentiated with IBC at indicated concentrations. On D5, cells were harvested and cell lysates were used for RNA extraction followed by cDNA synthesis and qPCR to analyze gene expression levels of *SREBP1c* (**A**); *adiponectin* (**B**); *ACC1* (**C**); and *FAS* (**D**). Data are presented as means ± SD of triplicate experiments. # *p* < 0.01 vs. control; * *p* < 0.01 vs. MDI only.

It is known that PPARγ, C/EBPα, and sterol regulatory element binding proteins1c (SREBP1c) transactivate genes related to lipid metabolism during adipogenesis [18]. Thus, we measured mRNA levels of *SREBP1c* and several downstream genes such as adiponectin, fatty acid synthase (*FAS*), and acetyl-CoA carboxylase-1 (*ACC1*). IBC decreased mRNA levels of *SREBP1c*, *adiponectin, ACC1*, and *FAS* in a dose-dependent manner compared to MDI treatment only (Figure 4). These data suggest that IBC might have an anti-obesity activity by regulating the expression of adipogenic genes.

### 2.5. IBC Inhibits Autophagic Flux during Adipocyte Differentiation

Autophagy plays a pivotal role in lipid formation in white adipocytes. During adipocyte differentiation, an increased number of autophagosome has been found under electron microscopy [19]. Autophagosome formation needs several autophagy related (Atg) proteins and microtubule-associated protein 1A/1B-light chain 3B (LC3B). LC3B-II (membrane localized lipidated form of LC3B) and SQSTM1/p62 (an autophagy adaptor protein) are associated with autophagosomal membranes. They are designed to fuse autophagosomes with lysosomes to form autolysosomes where cargoes are degraded.

**Figure 5:**
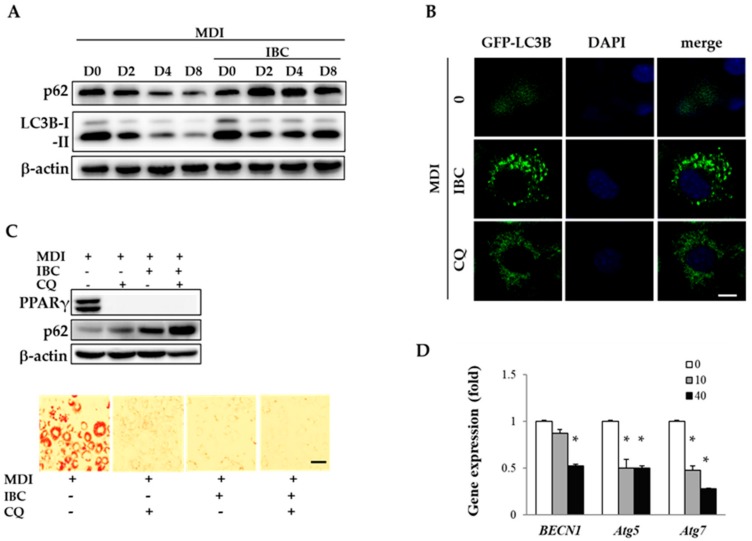
Effect of IBC on autophagic flux during adipocyte differentiation. (**A**) 3T3-L1 adipocytes were differentiated in the presence or absence of 40 μM of IBC. Differentiating adipocytes were harvested at the indicated period and lysed for Western blotting analysis to determine levels of LC3B and SQSTM1/p62; (**B**) Preadipocytes were transfected with GFP-LC3 plasmid and differentiated with MDI in the presence of IBC or CQ (10 μM) during D0–D2, followed by additional treatment with differentiation medium for 2 days (D4). Cells were fixed and stained with DAPI (nuclei, blue-colored). Intracellular GFP-LC3 puncta (green-colored) were visualized with a confocal laser microscope. Scale bar = 10 μm; (**C**) Differentiating adipocytes supplemented with IBC or CQ during D0–D2 were harvested and protein levels of PPARγ and SQSTM1/p62 were analyzed on D8. Scale bar = 100 μm; and (**D**) On D2, differentiating adipocytes supplemented with IBC or CQ were harvested and subjected to qPCR to analyze gene expression levels of *BECN1*, *Atg5*, and *Atg7*. Data are presented as means ± SD of triplicate experiments. * *p* < 0.01 vs. MDI only.

To understand whether autophagy contributes to the regulation of adipocyte differentiation by IBC, we observed the expression pattern of LC3B and SQSTM1/p62 as an autophagy marker by Western blotting analysis (Figure 5A). During adipocyte differentiation, protein levels of LC3B-I, LC3B-II, and SQSTM1/p62 were gradually reduced as evidence of autophagic flux. However, IBC treatment (40 μM) accumulated LC3BI, LC3B-II, and SQSTM1/p62 as compared to respective control cells from D2, indicating that autophagy flux was blocked at MCE stage. Autophagy flux can also be measured by observing the fluorescence of GFP-tagged LC3 puncta, which is widely used to visualize autophagy flux in cells [20]. We transfected 3T3-L1 preadipocytes with GFP-LC3 plasmid and treated with MDI in the presence of IBC or chloroquine (CQ, 10 μM) during D0–D2, followed by additional treatment with differentiation medium for 2 days (D4). Under confocal microscopy, a faint GFP-LC3 puncta was observed in adipocytes undergoing differentiation. However, a significant number of GFP-LC3 puncta appeared in the presence of CQ, an autophagy inhibitor. Consistent with data of Western blotting analysis showing increased levels of LC3B, IBC also induced GFP-LC3 puncta accumulation in the cytosol (Figure 5B), indicating that IBC interrupted autophagic flux.

To confirm the inhibitory effect of IBC on adipocyte differentiation, preadipocytes were differentiated in the presence of IBC or CQ during D0–D2 followed by ORO staining on D8. As shown in Figure 5C, treatment with IBC or CQ completely attenuated adipocyte differentiation. Combined treatment of IBC with CQ resulted in enhanced accumulation of SQSTM1/p62 compared with treatment with IBC or CQ alone. These data demonstrate that IBC treatment during the MCE period is sufficient to attenuate adipocyte differentiation via autophagy flux inhibition.

BECN1, a homolog of yeast Vps30/Atg6, and Atg proteins are key factors that initiate autophagosome formation. When the BECN1/Bcl-2 complex is disrupted by autophagy stimuli, BECN1 is consequently dissociated. It then contributes to autophagosome assembly. Deng et al. suggested that inhibition of autophagy by targeting BECN1 can affect obesity in mice [20]. In the present study, IBC decreased mRNA levels of *BECN1, Atg5*, and *Atg7* by 47.3%, 49.6%, and 71.8%, respectively, compared to MDI treatment alone (Figure 5D), indicating that IBC had an inhibitory effect on autophagosome formation. Although further studies are required to disclose the detailed action mechanism in different stages of autophagy, our results confirmed that IBC can inhibit adipocyte differentiation by interrupting autophagic flux in 3T3-L1 adipocytes.

### 2.6. IBC Inhibits Lipid Accumulation in High Cholesterol-Diet Zebrafish Larvae

**Figure 6:**
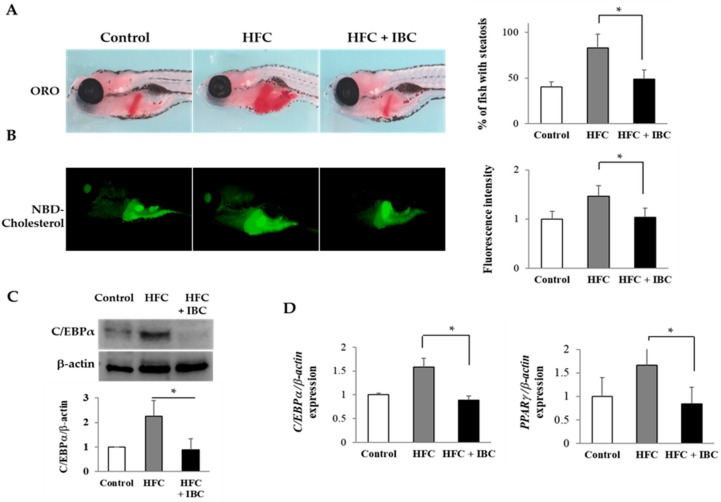
The anti-obesity effect of IBC in high fat cholesterol (HFC)-diet zebrafish. (**A**) Lateral view of 10 dpf larvae staining with ORO. Hepatic lipid accumulation was detected from HFC, and significant reduction of ORO staining was present in the liver with 100 nM IBC treatment (*n* = 75 each, * *p* < 0.01); (**B**) Representative images of live 10 dpf larvae following ingestion of NBD cholesterol (green-colored). Larvae with HFC showed high intensity in the gall bladder and intestine whereas larvae with the high fat cholesterol and 100 nM IBC treatment had reduced metabolism of NBD-cholesterol (*n* = 45 each, * *p* < 0.001); (**C**) Zebrafish total proteins were analyzed for C/EBPα expression. Data represent the mean ± SEM from 3 experiments (*n* = 30 each, * *p* < 0.01); and (**D**) Zebrafish total RNA was analyzed for *C/EBPα* and *PPARγ* expression. Data represent the mean ± SEM from 3 experiments (*n* = 30 each, * *p* < 0.05). Control, normal diet fed larvae; HFC, high fat cholesterol fed larvae; HFC + IBC, high fat cholesterol fed larvae with 100 nM IBC treatment.

To test the effect of IBC on the metabolism of zebrafish, 3 dpf embryos were fed with normal diet (Rotifers, Reed Mariculture Inc., Campbell, CA, USA), high fat cholesterol (HFC) diet and high fat cholesterol with 100 nM IBC for 10 days. To determine whether steatosis occurs in larvae, they were stained with ORO. Larvae fed with normal diet rarely stained ORO, whereas the incidence of steatosis was much higher in high fat cholesterol diet group. Interestingly, we observed that larvae fed with high fat cholesterol diet with 100 nM IBC did not develop steatosis (Figure 6A). Next, we assayed the effect of the IBC on the metabolism of fluorescent cholesterol analogues, NBD cholesterol, as determined by levels of biliary and intestinal fluorescence in live zebrafish larvae (Figure 6B). Larvae with the high fat cholesterol diet had intense gall bladder and intestinal fluorescence from lipase activity and the transport of phospholipids. However, IBC treatment reduced the fluorescence level of lipids in the high fat cholesterol treated larvae. These findings are compatible with the reduced steatosis in IBC treated larvae. Taken together, our results indicate that IBC plays a critical role in the development of steatosis and the inhibition of lipid absorption in larvae fed a high fat cholesterol diet.

To gain additional insights into the mechanism of action of IBC, we accessed its suppression effect on lipid metabolism in zebrafish. We analyzed mRNA and protein levels of transcription factors for adipogenesis by RT-PCR and Western blotting assay. The high fat cholesterol diet increased the transcription levels of *PPARγ* and *C/EBPα* as well in zebrafish. As shown in Figure 6C, IBC significantly suppressed the expression of C/EBPα in the high fat cholesterol-fed larvae. In addition, we found decreased mRNA levels of *C/EBPα* and *PPARγ* (Figure 6D) in IBC treated larvae in comparison with high fat cholesterol-fed larvae. These data confirm that IBC can rescue the phenotype of steatosis and obesity associated with high fat cholesterol treatment.

## 3. Discussion

Mouse preadipocyte 3T3-L1 is a well-established cell line to study the regulatory effect of various chemicals on adipogenesis and adipolysis [21]. Hormonal stimulation with 3-isobutyl-1-methylxanthine, dexamethasone, and insulin (MDI) can initiate adipocyte differentiation (also called adipogenesis) accompanied by the conversion of preadipocytes into mature adipocytes. At the early stage of adipogenesis, confluent preadipocytes undergo several rounds of cell division referred to as mitotic clonal expansion (MCE) after MDI stimulation followed by growth arrest. Therefore, cell cycle regulation at the early stage of adipogenesis has been considered as a strategy for modulating adipogenesis. Recently, many natural products have been reported to be anti-obesity agents that can inhibit MCE of 3T3-L1 adipocytes [2]. Widdrol and coagulanolides can arrest 3T3-L1 cells in the G1/S phase, while rohitukine can cause cell arrest in S/G2 [22,23,24]. Garcinol and pterostilbene can induce G2/M arrest by modulating the cell cycle regulators [25].

In this study, we found that IBC isolated from roots of edible plant *Angelica keiskei* (AK) could attenuate cell cycle progression through G0/G1 arrest during MCE by decreasing protein levels of cyclin D1, B1, cyclin-dependent kinase (cdk)-4, and cdk-6 (Figure 2). Among natural compounds, curcumin, resveratrol and sulforaphane have been reported to be able to block MCE in the G0/G1 phase by decreasing cyclin D1, cyclin E1, cdk-4, or cdk-6 expression [17,26,27]. Since the formation of the cyclinD1-cdk4/6 complex is required for post-confluent preadipocytes to enter cell cycles, decreased protein levels of cyclin D1, B1, cdk-4, and cdk-6 might have contributed to the inhibition of MCE by IBC during adipogenesis (Figure 2C).

After MCE, master adipogenic transcriptional factors, PPARγ and C/EBPα cooperatively express lipogenic genes [28]. PPARγ is a member of nuclear receptor belonging to PPARs family. It is expressed specifically in adipose tissue to modulate the expression of genes with critical roles in differentiation. C/EBPα is also an important transcription factor required for terminal adipogenesis. Sterol regulatory element-binding transcription factor (SREBP) is another master regulator of lipid metabolism. Among SREBP isoforms (SREBP1a, 1c, and 2), SREBP1c is abundant in adipose tissue as a mediator of lipogenesis by insulin [29].

Consistent with previous studies, mRNA and protein levels of PPARγ and C/EBPα were strongly increased by MDI stimulation. IBC significantly down-regulated PPARγ and C/EBPα expression that contributed to the suppression of adipocyte differentiation (Figure 3).

Many lipogenesis related genes are targets of PPARγ, C/EBPα, and SREBP1c [18,30]. Adiponectin acts as one of adipokines that can enhance adipocyte differentiation and lipid accumulation in mature adipocytes [31]. Acetyl-CoA carboxylase (ACC)-1 catalyzes fatty acid synthesis and contributes to lipid accumulation and adipogenesis in adipocytes [32]. Fatty acid synthase (FAS) is predominantly expressed in adipose tissue. It can induce intracellular lipid accumulation via catalysis of saturated fatty acids synthesis [33]. These downstream genes have been considered as terminal markers of adipocyte differentiation. As shown in Figure 4, IBC reduced mRNA levels of *SREBP1c*, *adiponectin*, *ACC-1*, and *FAS* to inhibit adipogenesis, suggesting its potential anti-obesity effect.

Autophagy is a highly orchestrated process to maintain several physiological activities, including the following: (1) Degradation of intracellular macromolecules and organelles via the lysosomal system, (2) clearance of disused proteins and organelles, pathogens, and deleterious proteins, and (3) regulation of energy status. Dysfunction of autophagy provokes the risk of cancer, myopathies, neurodegeneration, heart failure, and liver failure [34]. Autophagy is a well-known process that affects glucose and lipid metabolism in the obese state. Omental and subcutaneous adipose tissues in obese individuals represent elevated autophagic gene expression. Recent studies have shown that obese individuals and animals have excessive autophagic flux [35,36]. Genetic aberration of autophagy related genes (Atg) such as Atg5 and Atg7 can block adipogenesis [37,38]. Skop et al. proved that autophagy is required for differentiation of 3T3-L1 adipocytes [39]. They showed that the treatment of autophagic inhibitor, l-asparagine, during MCE stage could suppress the adipocyte differentiation process. Therefore, blocking autophagic flux by IBC treatment might lead to the inhibition of MCE, thus attenuating adipogenesis.

Recently, several attempts have been made to find autophagy regulators for treating obesity and metabolic diseases. For example, berberine, a noted phytochemical, can decrease autophagy flux with the anti-obesity effect by destabilizing BECN1 [20]. At the autophagy initiation step, BECN1 plays a role in the construction of the autophagy machinery. When the BECN1/Bcl-2 complex is dissociated, free BECN1 can form a complex with Atg proteins (including Atg5 and Atg7) and Atg-related proteins to promote assembly and elongation of the autophagosome membrane. Sequentially, cytosolic lipidated LC3B-II is incorporated into the autophagosomal membrane for mature autophagosomes [40]. Zhang et al. [41] observed autophagy activation during adipocyte differentiation. The autophagy flux during adipogenesis was confirmed by showing a decreased p62 level and LC3B-II/LC3B-I ratio, and these were reversed by an autophagy inhibitor (chloroquine, CQ). CQ suppressed the PPARγ expression to inhibit adipogenesis. Consistent with the previous findings, we observed a decreased p62 level and LC3B-II/LC3B-I ratio, and these were reversed by IBC treatment during early stage (D0–D2) of adipogenesis (Figure 5). IBC also suppressed the expression of *BECN1, Atg5*, and *Atg7* that are essential for autophagosome assembly. These data demonstrate that the inhibitory effect of IBC on autophagy flux contributes to suppression of adipocyte differentiation and autophagosome formation. Since pharmacological modulators of autophagy have been considered as potential drug candidates for treating autophagy-related diseases, our results might provide significant insights into the development of novel anti-obesity drugs [42].

In the present study, we used a high cholesterol diet-induced obesity zebrafish model to evaluate the anti-obesity effect of IBC in vivo [43]. Zebrafish are useful to study glucose and lipid metabolic disorders such as obesity, hyperglycemia, and diabetes due to their short life cycle and assembled full genome sequences. Lipid metabolism pathways and functions of adipose tissue including insulin homeostasis, secretion of endocrine adipokines, and fat storage are conserved between fish and humans [44,45]. It has been reported that natural compounds such as cholecalciferol, tanshinone IIA, and astaxanthin are potential protective agents against obesity or nonalcoholic steatohepatitis in zebrafish model [46,47,48]. As shown in Figure 6, zebrafish fed a high fat cholesterol diet showed increased intrahepatic fat deposits and steatosis (indicators of obese status). However, zebrafish fed a high cholesterol diet supplemented with IBC showed reduced fat accumulation in the liver as well as intestine. Moreover, decreased levels of lipogenic markers PPARγ and C/EBPα after treatment with IBC confirmed the anti-obesity effect of IBC. The anti-obesity effect of IBC from 3T3-L1 cells is consistent with zebrafish data.

Although many attempts have been made to develop new anti-obesity drugs, the obesity rate continues to increase worldwide. As natural compounds combined with pharmacologic or non-pharmacologic treatment are effective for treating obesity, we need to continuously explore new anti-obesity compounds. For that reason, we have focused on herbal constituents including food ingredients.

In this study, we isolated IBC from the edible herb *A. keiskei* as a promising anti-obesity agent. It can inhibit lipid accumulation in adipocytes and zebrafish. Our results suggest that suppression of autophagy flux and MCE is an underlying mechanism of IBC.

## 4. Materials and Methods

### 4.1. Isolation of Isobavachalcone (IBC) from Root of Angelica keiskei

Air-dried roots of *A. keiskei* were extracted with ethanol and evaporated to dryness. These extracts were dissolved in water and partitioned with ethyl acetate. IBC was purified from ethyl acetate soluble fraction and the structure was elucidated by the spectroscopic data analysis and comparison with previously described data [49].

### 4.2. Culture and Preadipocyte Differentiation

3T3-L1 preadipocytes were obtained from American Type Culture Collection (Manassas, VA, USA). They were cultured in growth medium (DMEM, WelGENE, Daegu, Korea) supplemented with 10% newborn calf serum (Gibco BRL Life Technology, Grand Island, NY, USA) without antibiotics at 37 °C with 5% CO_2_-air atmosphere. After cells reached 100% confluence (about two days of culture), the culture medium was replaced with differentiation medium containing MDI (isobutyl-methylxanthine 1 µg/mL, dexamethasone 1 µM, and insulin 1 µg/mL) in DMEM (differentiation day 0: D0). Two days later (D2), differentiation media were replaced with insulin-containing DMEM. After two days of incubation (D4), cells were maintained in DMEM with 10% fetal bovine serum (FBS, Lonza, Walkersville, MD, USA) and cultured for additional 4 days (D8). IBC was dissolved in DMSO and added to these cells at indicated concentrations during the differentiation period.

### 4.3. MTT Assay and Oil Red O (ORO) Staining

To determine the effect of IBC on cell proliferation, 3T3-L1 cells were plated into 24-well plates and differentiated in the presence or absence of IBC. On D0, D2, or D8, cells were treated with 3-(4,5-dimethylthiazol-2-yl)-2,5-diphenyltetrazoliumbromide (MTT) (Sigma, St. Louis, MO, USA) solution at 37 °C. After 3 h of incubation, MTT solution was removed and 100 µL of DMSO was added to each well to solubilize MTT formazan crystals. The absorbance was then measured at wavelength of 570 nm using a GloMax^®^-Multi Microplate Multimode Reader (Promega, Madison, WI, USA).

The lipid content in differentiated 3T3-L1 cells was evaluated by Oil red-O (ORO) staining. Cells were washed with phosphate buffered saline (PBS), fixed in 10% formalin in PBS for 1 h at 4 °C, washed with PBS twice, and stained with 0.5% ORO in 60% isopropanol for 30 min at 4 °C. To quantify the intracellular lipid content, excess stain was removed by washing with 70% ethanol and cells were extracted with 4% Nonidet P-40 in isopropanol. The absorbance of the extract solution was then measured at a wavelength of 520 nm on a microplate reader. Images of accumulated lipid drops in mature adipocyte were obtained using an inverted phase-contrast microscope (TH4, Olympus, Tokyo, Japan).

### 4.4. Flow Cytometry

To measure cell cycle progression, confluent 3T3-L1 adipocytes were incubated with MDI in the presence or absence of IBC (40 μM) for 48 h and subjected to flow cytometry after staining with propidium iodide (PI) (Sigma). Briefly, cells were washed with PBS twice, fixed with cold-absolute ethanol for 1 h at 40 °C, incubated at 37 °C with 50 μg/mL of RNase for 1 h, and stained with 40 μg/mL of PI for 30 min at room temperature. Approximately 10, 000 cells per each sample were analyzed using FACS Calibur system (Becton Dickinson, San Jose, CA, USA). Cell cycle progression was analyzed with the CELL Quest program.

### 4.5. Transfection and Confocal Microscopy

3T3-L1 preadipocytes were grown on a 35-mm glass bottom confocal dish (SPL Life Sciences, Gyeonggi-do, Korea) and transiently transfected with GFP-LC3B plasmid (kindly provided by Professor Keun-Il Kim, Sookmyung Women’s University, Seoul, Korea) using Lipofectamine^®^ 2000 Reagent (Invitrogen, Carlsbad, CA, USA). Two days after the transfected cells became confluent, cells were treated with MDI in the presence or absence of IBC. On D2, cells were fixed with 2% paraformaldehyde for 5 min, washed with PBS, and stained with DAPI (4′,6-diamidino-2-phenylindole, Sigma, St. Louis, MO, USA). Intracellular GFP-LC3B puncta were detected by an LSM 700 confocal laser microscope (Carl Zeiss, Oberkochen, Germany).

### 4.6. Zebrafish Experiments

All zebrafish (Zebrafish International Resource Center, Eugene, OR, USA) husbandry and experimental protocols complied with institutional guidelines and were approved by local ethics boards (Sookmyung Women’s University Animal Care and Use Committee, SMWU-IACUC-1712-036). Adult zebrafish were maintained under standard conditions at 28.5 °C with a 14 h light/10 h dark cycle [50]. Embryos were obtained from natural crosses between wild type AB strain fish. Zebrafish embryos (3 dpf) were fed twice a day in embryonic water containing a standard diet (rotifers and dietary pellets) or high fat cholesterol diet for 10 days. IBC was dissolved in DMSO and added to the embryonic water and changed daily. To make the high fat cholesterol diet, cholesterol (Sigma) solution in diethyl ether was absorbed into standard food (Tetramin: Crude lipid 11%, omega-3 fatty acids 500 mg/kg, Tetra Werke, Melle, Germany), evaporated and grounded to make fine particles [51].

### 4.7. Whole Mount ORO Staining and NBD Cholesterol Staining

Zebrafish larvae were fixed in 4% paraformaldehyde, washed with 60% isopropanol and incubated with 0.3% ORO in 60% isopropanol [52]. Stained larvae were imaged on a bright-field dissecting microscope (Nikon SMZ1500, Tokyo, Japan). To study cholesterol uptake in live larvae, zebrafish larvae (13 dpf) were starved for 6 h and soaked in NBD cholesterol (3 mg/mL, solubilized with fish bile, Invitrogen) for 2 h and observed under a fluorescent microscope (Olympus IX71) [43]. All images were analyzed with the ImageJ program (NIH, Bethesda, MD, USA).

### 4.8. RNA Extraction and Quantitative Real Time-PCR (qRCR)

Cultures of 3T3-L1 preadipocytes were plated at a density of 2.5 × 10^5^ cells into a 60-mm dish and differentiated as mentioned above. Cells were lysed with TriZol reagent (Molecular Research Center, Cincinnati, OH, USA) at D5 to purify total RNA which was then used for cDNA synthesis (Labopass™ cDNA synthesis kit, Cosmogenetech, Seoul, Korea). Then, cDNA was used to estimate gene expression level during adipocyte differentiation by qPCR using SYBR Premix Ex TaqTM real time PCR Kit (Cosmogenetech) and Applied Biosystems 7500 Fast Real-Time PCR System (Foster City, CA, USA). The glyceraldehyde 3-phosphate dehydrogenase (GAPDH) mRNA level was used as an internal control to determine the relative mRNA level of adipogenic factors. Primers used for amplifications are listed in Table 1.

### 4.9. Western Blot Analysis

Cells were harvested and resuspended in lysis buffer (25 mM Tris-Cl, pH 7.5, 100 mM NaCl, 1% NP-40, 1% sodium deoxycholate, 0.1% sodium dodecyl sulfate and protease inhibitor cocktails) followed by centrifugation at 15,000 rpm for 20 min. Total proteins (40 μg) were loaded onto sodium dodecyl sulfate-polyacrylamide gel, electrophoresed, and transferred to PVDF membrane. The membrane was probed with primary antibody for PPARγ (Cell Signaling Technology, Danvers, MA, USA), C/EBPα (Cell Signaling), cyclin D1 (Cell Signaling), cyclin B1 (Cell Signaling), cdk-4 (Abcam, Cambridge, UK), cdk-6 (Cell Signaling), LC3B (Cell Signaling), or SQSTM1/p62 (Sigma Aldrich). It was then incubated with HRP-conjugated anti-mouse (Santa Cruz, Dallas, TX, USA) or anti-rabbit IgG (Santa Cruz) secondary antibody. Immunoreactivities of proteins were visualized with an Enhanced Chemiluminescence detection system (Bio-Rad, Hercules, CA, USA). Protein levels were quantified using the Fusion Solo system (Vilber Lourmat, Collegien, France). β-Actin (Sigma) served as a loading control.

### 4.10. Statistical Analysis

All values are presented as mean ± standard deviation. Differences were assessed using Student’s *t*-test or one-way analysis of variance (ANOVA) followed by Dunnett’s test. All experiments were performed at least three times. Differences with a *p* value of less than 0.05 were considered statistically significant.

## Figures and Tables

**Table 1:** Oligonucleotide primer sequences used for the qRT-PCR analysis.

Gene Name	Forward Primer	Reverse Primer	Accession Number
*PPARγ*	AACTCTGGGAGATTCTCCTGTTGA	GAAGTGCTCATAGGCAGTGCAT	EF062476
*PPARγ(Zf)*	AGTACGGGGTCATCGAAGTG	GCGCAGACTCTTGAGGAACT	NM131467.1
*C/EBPα*	TGCACCACCAACTGCTTAG	AAACCATCCTCTGGGTCTCC	NM001287523
*C/EBPα(Zf)*	CATCGACATCAGCGCCTACA	CACCGTGGTGGTAGTCGTAG	NC007118.7
*SREBP1c*	TGTTGGCATCCTGCTATCTG	AGGGAAAGCTTTGGGGTCTA	XP011247147.
*Adiponectin*	TGTAGGATTGTCAGTGGATCTG	GCTCTTCAGTTGTAGTAACGTCATC	NP033735.3
*FAS*	AGCGGCCATTTCCATTGCCC	CCATGCCCAGAGGGTGGTTG	NP032014.3
*ACC1*	GTCAGCGGATGGGCGGAATG	CGCCGGATGCCATGCTCAAC	XP011247147.1
*BECN1*	ACCGGGTCACCATCCAGGAA	GAAGCTATTAGCACTTTCTGT	NP062530.2
*Atg5*	TGTGCTTCGAGATGTGTGGTT	GTCAAATAGCTGACTCTTGGCAA	NP444299.1
*Atg7*	CCTGCACAACACCAACACAC	CACCTGACTTTATGGCTTCCC	NP001240646
*GAPDH*	TGCACCACCAACTGCTTAG	GGCATGGACTGTGGTCATGAG	BC096042

*PPARγ*, peroxisome proliferator activated receptor subtype gamma; *C/EBPα*, CCAAT/enhancer binding protein-alpha; *SREBP1c*, sterol regulatory element-binding proteins 1c; *FAS*, fatty acid synthase; *ACC1*, acetyl-CoA carboxylase-1; *Atg5* and *Atg7*, autophagy related gene 5 and 7; *GAPDH*, glyceraldehyde 3-phosphate dehydrogenase; *PPARγ2* (Zf), PPARγ primer used in zebrafish; *C/EBPα* (Zf), C/EBPα primer used in zebrafish.
